# Optimization of the Passivation Process for AM 350 and CUSTOM 450 Stainless Steels Using Taguchi Methodology and Gray Relational Analysis

**DOI:** 10.3390/ma19091846

**Published:** 2026-04-30

**Authors:** Facundo Almeraya-Calderon, Jose Cabral-Miramontes, Miguel Villegas-Tovar, Demetrio Nieves-Mendoza, Erick Maldonado-Bandala, María Lara-Banda, Brenda Paola Baltazar-Garcia, Oliver Samaniego-Gamez, Ce Tochtli Méndez-Ramírez, Javier Olguin-Coca, Citlalli Gaona-Tiburcio

**Affiliations:** 1 Universidad Autónoma de Nuevo Leòn, FIME, Centro de Investigación e Innovación en Ingeniería Aeronáutica (CIIIA), eón, San Nicolás de los Garza 66455, Mexico; facundo.almerayacld@uanl.edu.mx (F.A.-C.); miguel.villegastvr@uanl.edu.mx (M.V.-T.); maria.laraba@uanl.edu.mx (M.L.-B.); citlalli.gaonatbr@uanl.edu.mx (C.G.-T.); 2 Facultad de Ingeniería Civil, Universidad Veracruzana, Xalapa 91000, Mexico; dnieves@uv.mx (D.N.-M.); erimaldonado@uv.mx (E.M.-B.); pao.baltazar.08@gmail.com (B.P.B.-G.); 3 Instituto de Ingeniería y Tecnología, Universidad Autónoma de Ciudad Juárez, Av. del Charro 450, Partido Romero, Ciudad Juárez 32310, Mexico; oligmez94@gmail.com; 4 Área Académica de Ingeniería y Arquitectura, Universidad Autónoma del Estado de Hidalgo, Carretera Pachuca-Tulancingo Km. 4.5, Pachuca de Soto 42082, Mexico; olguinc@uaeh.edu.mx

**Keywords:** corrosion resistance, conversion coatings, protection, aeronautical

## Abstract

This study presents research on optimizing the parameters of the passivation process for precipitation-hardening stainless steels (PHSS) to improve the corrosion resistance of AM350 and CUSTOM 450 alloys, which are extensively utilized in the aerospace and aviation sectors, since, as this is a complex process, it requires the implementation of a robust methodological approach that allows for multi-response optimization. Experiments were designed using the Taguchi method, which offered a strong framework for examining the impact of material, type of passivation solution, concentration, temperature, and passivation process time on the corrosion resistance of both PHSS alloys. To confirm the ideal PHSS passivation process parameters and measure the significance of each component, gray relational analysis (GRA) and analysis of variance (ANOVA) were also employed. The combined use of the Taguchi/GRA represents a robust and efficient methodological approach to the multi-response optimization of complex processes, overcoming the limitations inherent in the individual application of each technique. It was determined that the optimized parameters were a PHSS AM 350, a solution composed of a combination of citric acid and oxalic acid, acid concentration of 25% *v*/*v*, temperature of 50 °C, and time of 120 min. This combination of parameters resulted in significant improvements of up to 55% in corrosion resistance in the H_2_SO_4_ and NaCl evaluation solutions, demonstrating the effectiveness of the optimized conditions. This work emphasizes the efficacy of integrating Taguchi, GRA, and ANOVA techniques to significantly reduce the corrosion rate of PHSS undergoing the passivation process using alternatives to nitric acid. The integration of the Taguchi methodology with GRA enables the normalization and combination of responses with different scales and performance criteria into a single gray relational index, facilitating the overall evaluation of the system.

## 1. Introduction

The aerospace industry is one of the most demanding sectors in terms of material quality, safety, and performance, where each component must meet rigorous standards for mechanical strength, corrosion resistance, and durability under extreme operating conditions [1,2]. In this context, high-strength stainless steels have emerged as essential materials for critical applications, particularly in structural systems, landing gear, engine components, and fasteners that must withstand high loads and corrosive environments [3,4]. Among these materials, AM350 and CUSTOM 450 stainless steels stand out for their exceptional combination of higher mechanical strength, fracture toughness, and corrosion resistance, characteristics that make them preferred choices for high-performance aero-nautical components [5,6].

AM350 stainless steel, classified as a precipitation-hardening semi-austenitic steel, offers tensile strengths exceeding 1300 MPa after appropriate heat treatments, while maintaining excellent toughness and fatigue resistance [7]. CUSTOM 450, also known as martensitic precipitation-hardening stainless steel, has comparable strength with superior stress corrosion resistance and is widely used in applications requiring optimal combinations of strength and resistance to marine or corrosive environments [8]. However, despite their inherent properties, these steels require appropriate surface treatments to maximize their anti-corrosion performance, with chemical passivation being the most used process for this purpose [9,10].

Passivation is a chemical process that involves the elimination of free iron from the surface of stainless steel and the enhancement of a chromium-enriched passive layer, which provides protection against corrosion [11]. This process involves multiple parameters that must be carefully controlled, including the type of passivating medium (nitric acid, citric acid, among others), the primary material, the concentration of the chemical agent, the operating temperature, and the immersion time [12,13]. The optimization of these parameters is critical to achieving maximum corrosion resistance without compromising the integrity of the material or generating unnecessary operating costs [14].

To address the complexity inherent in multiparametric optimization of the passivation process, robust statistical and data analysis methodologies have been employed to obtain valuable information with experimental efficiency. One of the most important experimental design approaches in contemporary quality engineering is the Taguchi method [15,16]. This methodology is based on the philosophy of “robust design,” which seeks to create products and processes that are insensitive to noise factors or uncontrollable environmental variables, thus ensuring consistent performance under variable operating conditions [17,18].

The foundation of the Taguchi approach is its capacity to use orthogonal arrays to systematically examine the impact of several factors on one or more response variables using a significantly smaller number of tests, also known as orthogonal matrices [19]. These orthogonal arrays, designated by the nomenclature L_n(m^k^), where *n* is the number of experiments, *m* is the number of levels of each factor, and *k* the number of factors that can be studied, allow for a balanced distribution of experimental combinations, ensuring that each level of each factor appears the same number of times and that the effects of the factors can be separated independently [20,21]. For example, an L9(3^4^) arrangement allows up to four factors with three levels each to be studied using only nine experiments, in contrast to the 81 runs that a complete factorial design would require [22].

The Taguchi methodology also incorporates the concept of signal-to-noise ratio (S/N), a metric that simultaneously quantifies the meaning and variability of the quality characteristic under study [15,23]. Different S/N ratio types are employed, depending on the optimization aim: “Larger-the-better” for characteristics that are to be maximized, “Smaller-the-better” for characteristics that are to be minimized, and “Nominal-the-best” for characteristics with a specific target value [18,24]. This transformation allows multiple replicates to be converted into a single value that reflects both the average performance and the consistency of the process, facilitating the identification of optimal process conditions that minimize variability and maximize the quality of the final product [25].

Numerous areas of materials engineering and manufacturing processes have shown the Taguchi method’s applicability. One example is where the Taguchi L18 design was used to optimize cutting parameters in the steel turning process, investigating the effect of cutting speed, feed rate, cutting depth, and angle of incidence on surface finish. The findings allowed for the determination of the ideal set of parameters that reduced surface roughness, reducing R_a_ values from 1.94 μm to 0.58 μm, demonstrating the effectiveness of the methodology in significantly improving process quality with a small number of experiments [24]. In field of metal surface treatments, they applied Taguchi L27 method to optimize the 6061-aluminum anodizing process, studying the effects of sulfuric acid concentration, current density, electrolyte temperature, and treatment time on the thickness of the anodic oxide layer and corrosion resistance. Through signal-to-noise ratio analysis and subsequent experimental validation, the researchers established optimal conditions that increased corrosion resistance by 45% compared to conventional conditions, demonstrating the potential of this methodology to improve surface protection treatments like passivation [21].

Furthermore, analysis of variance (ANOVA), a basic statistical method, enables us to quantify the percentage contribution of each parameter to the outcome and ascertain the statistical significance of the factors under investigation and their interactions on the response variables [22,26]. This methodology has evolved to become one of the best versatile and generally used statistical tools in scientific investigation and experimental engineering [27,28]. The fundamental principle of ANOVA lies in the partitioning of the total variability observed in the experimental data into components attributable to different sources as follows: variability due to controlled factors, variability due to interactions between factors, and random variability or experimental error [29,30].

The mechanics of ANOVA are based on comparing the means of different groups by calculating the F-statistics, which represents the ratio between the variance justified by the factor and the variance of the error [22]. When the calculated F value exceeds the critical F-value for a predetermined significance level (typically α = 0.05 or α = 0.01), the factor has a statistically significant impact on the response variable, as indicated by the rejection of the null hypothesis that all group averages are equal [31,32]. Additionally, ANOVA provides quantitative information on the magnitude of each factor’s effect by calculating the percentage contribution, which is obtained by dividing the sum of squares for each factor by the total sum of squares; this allows the factors to be ranked according to their relative importance in the process under study [18,20]. This tool is essential for validating Taguchi design findings and establishing reliable cause-and-effect relationships in complex experimental systems, providing the statistical rigor necessary for evidence-based decision-making [17,27]. While the Taguchi method identifies optimal process conditions by analyzing signal-to-noise ratios, ANOVA complements this analysis by statistically confirming which factors have real effects on the process and which can be attributed merely to random variability [16]. This statistical validation is particularly critical in industrial applications where process optimization decisions involve significant capital investments or have direct consequences on product safety and reliability, as is the case with aeronautical components [33].

For example, in the field of chemical metal treatments, they applied the Taguchi methodology mixed with ANOVA to optimize the electropolishing process of 316L stainless steel, a material widely used in biomedical and food processing applications. The L16 experimental design included factors such as phosphoric acid concentration, sulfuric acid concentration, current density, temperature, and treatment time, analyzing their effect on surface roughness and the rate of material removal. The ANOVA analysis revealed that the current density (38.6% contribution) and temperature (29.4% contribution) were the most significant factors (*p* < 0.01), while treatment time had a marginal effect (4.2% contribution, *p* > 0.05). This information made it possible to simplify process control by focusing on critical parameters and relaxing tolerances on less influential parameters, thereby reducing production costs without compromising treatment quality [34].

However, when it is necessary to simultaneously optimize multiple quality characteristics that may present conflicting or contradictory objectives, gray relational analysis (GRA) emerges as a particularly valuable and versatile methodology [35,36]. This situation is common in complex industrial processes where, for example, maximizing corrosion resistance could involve prolonged treatment times that increase operating costs, or were improving one surface property could compromise another equally important characteristic [37,38]. GRA, based on the gray systems theory represents an innovative mathematical approach that allows multi-objective optimization problems to be changed into a single-objective optimization problem by calculating Gray Relational Coefficients (GRCs) and gray relational grades (GRGs), facilitating rational decision-making in systems characterized by incomplete, uncertain, or partially known information [35,39,40].

The gray system theory is based on the concept that real-world systems can be classified according to the degree of available information as follows: totally known information (white systems), totally unknown information (black systems), and partially known information (gray systems) [41,42]. Most industrial processes and engineering phenomena belong to the category of gray systems, where some experimental information is available, but it is impractical or impossible to fully characterize all variables and their interactions [36,43]. GRA addresses this inherent uncertainty by analyzing the geometric similarities between data sequences, establishing quantitative relationships between a reference sequence representing ideal or desired performance and multiple comparative sequences representing experimental results obtained under different conditions [39,44].

The application of GRA has proven effective in numerous manufacturing and material treatment processes. In a study, researchers aimed to optimize the hard coating process using gas tungsten arc welding (GTAW) on low-carbon steel, evaluating the deposition of nickel-based alloys for applications in components subject to severe wear. The researchers used GRA to simultaneously optimize the following six coating characteristics: hardness (maximize), deposition rate (maximize), dilution (minimize), surface roughness (minimize), porosity (minimize), and contact angle (specific target of 45°). The L27 experimental design investigated the influences of welding current, welding rate, electrode-to-workpiece distance, and shielding gas flow speed. The GRA results, with a maximum GRG of 0.912, identified optimal conditions that simultaneously increased coating hardness by 27% (from 412 HV to 523 HV), reduced dilution by 35% (improving metallurgical quality), and decreased porosity by 42%, proving the approach’s capacity to resolve difficult multi-objective optimization problems where conventional univariate optimization methods would have failed to find the optimal balance [45].

Using Taguchi/GRA/ANOVA methodologies in combination provides a robust, efficient, and cost-effective multi-response optimization approach, clearly overcoming the limitations of using each technique individually. This synergy makes it possible to obtain optimal overall process conditions with greater reliability and less experimental effort. This research represents a significant contribution to scientific and technological knowledge in the field of surface treatments for aeronautical stainless steels, integrating advanced statistical methodologies for the systematic optimization of the passivation process in AM350 and CUSTOM 450 alloys. Highlights include the identification of optimal passivation conditions specific to each alloy, considering multiple performance criteria simultaneously, the quantification of the individual contribution of the individual process parameter to treatment quality, and the establishment of methodological bases that can be extrapolated to other material systems and surface treatment processes in the aerospace industry.

## 2. Materials and Methods

### 2.1. Materials

For this study, cylindrical bars with a radius of 1.25 cm and a thickness of 1.00 cm were used, made from commercial precipitation-hardening stainless steel (PHSS) of grades AM 350 and CUSTOM 450. These materials are martensitic precipitation-hardening stainless steels. Table 1 displays the chemical composition of both materials.

PHSS was prepared using metallographic technique, by means of 120, 240, 400 and 600 grit sandpaper (silicon carbide) prior to the passivation process [46]. After cleaning each sample with deionized water, they were ultrasonically treated in ethanol for ten minutes.

### 2.2. Chemical Passivation Treatment

The recommendations of ASTM A967-17 [47] were followed to passivate the steels under study. The treatment was as follows:

1. In accordance with ASTM A380-17 and SAE AMS 2700F standards, the PHSS samples were washed with ultrasound for ten minutes in ethanol and then submerged in deionized water [48,49].

2. The four types of passivation baths were:

(a) citric acid (C_6_H_8_O_7_) at concentrations of 15 and 25% weight/volume (% *w*/*v*) by volume;
(b) combination of citric acid and oxalic acid at two different concentrations—the first being 15% weight/volume (% *w*/*v*) citric acid with 10% *w*/*v* oxalic acid for a total of 25% volume/volume (% *v*/*v*), and the second being 25% weight/volume (% *w*/*v*) citric acid with 10% weight/volume (% *w*/*v*) oxalic acid for a total of 35% volume/volume (% *v*/*v*);
(c) a combination of citric acid and hydrogen peroxide with two different concentrations—the first being 15% weight/volume (% *w*/*v*) citric acid with 10% volume/volume (% *v*/*v*) hydrogen peroxide for a total of 25% volume/volume (% *v*/*v*), and the second being 25% weight/volume (% *w*/*v*) citric acid with 10% weight/volume (% *w*/*v*) hydrogen peroxide for a total of 35% volume/volume (% *v*/*v*);
(d) a combination of citric acid, hydrogen peroxide, and ethanol with two different concentrations: 15% weight/volume (% *w*/*v*) citric acid with 10% volume/volume (% *v*/*v*) hydrogen peroxide and 5% volume/volume (% *v*/*v*) ethanol for a total of 30% volume/volume (% *v*/*v*) and the second with 25% volume/volume (% *v*/*v*) citric acid with 10% volume/volume (% *v*/*v*) hydrogen peroxide and 5% volume/volume (% *v*/*v*) ethanol for a total of 40% volume/volume (% *v*/*v*).

3. The temperature of the passivation solutions varied between 25 and 50 °C.

4. The duration of the immersion ranges from 90 to 120 min.

5. The treatment was completed with air drying and rinsing with deionized water [50].

**Figure 1:**
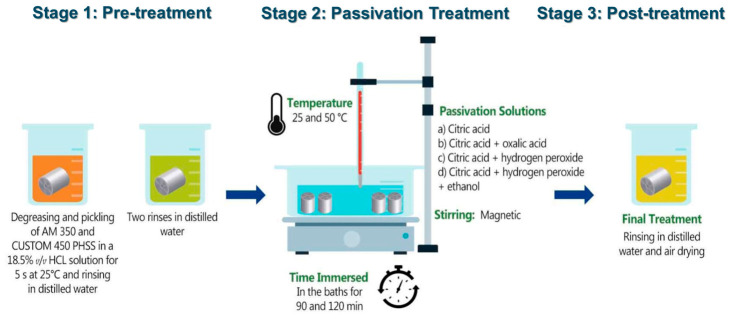
Experimental diagram of the Chemical Passivation Treatment.

Figure 1 shows the experimental diagram, which outlines the different phases involved in the passivation process. Table 2 shows the parameters used for the passivation process of PHSS in detail.

### 2.3. Design of Experiments (DOE)

In investigation development and industrial applications, the design of experiments (DOEs) is crucial because it offers an organized method for methodically examining how various elements affect desired results. DOE makes it possible to efficiently investigate correlations between variables, optimize processes, and enhance product quality [51]. This is especially relevant in process optimization with the passivation of stainless steel. In this work, DOE is used as a framework to estimate the influence of passivation process parameters, such as bath chemical composition (A), alloy chemical composition (B), bath concentration (C), temperature (D), and time (E), on the electrochemical properties of the passive layer. Therefore, in this research work, these five factors are considered as design parameters, with four levels for the passivating bath solution (A) and two levels for the rest of the factors (B–E) in conjunction with their interactions. Table 3 shows the design factors and their levels. To optimize the corrosion resistance of the passivated coating in solutions of H_2_SO_4_ and NaCl solutions, the tests were designed using Taguchi’s L16 mixed orthogonal array (MOA) [52] (pp. 762–783), and the results were analyzed using Minitab^®^ 21 software [53]. Table 4 summarizes the parameter combinations selected for the experiment, describing the input parameters and their levels.

A manageable scope for the investigation was ensured by keeping several parameters constant to maintain the integrity of the experimental design. We were able to concentrate on the crucial elements of the passivation process of precipitation-hardening stainless steels that influence corrosion resistance in the two test solutions previously indicated because this method reduced the number of trials and the complexity of the study [54]. We were able to better understand the elements influencing the quality of the passive layer created on stainless steels by optimizing the design and increasing the reliability of the results [15]. The parameters specified in the experimental design were followed while preparing the passivation coatings, and the chosen conditions were given at random to reduce systematic error in the experiment [52] (pp. 792–793). This randomization increases the dependability of the results and guarantees that no uncontrolled variables will skew the experimental outcomes [55]. As previously stated, the Taguchi technique reduces the number of tests required to determine the ideal process parameters by using an MOA. With the fewest experimental trials possible, MOA can be used to calculate the main and interaction effects [16]. The number of design factors and how they interact determine which MOA is best. In this case, a MOA design was used because one of the factors has four levels; an MOA L16 is selected, which has 16 rows that match to the number of trials and 15 degrees of freedom (DOFs) with 13 columns—12 columns for factors with two levels and the first column for factors with four levels (4^1^ 2^4^) [56]. The table for MOA is used to assign the components and their most significant interactions to the matrix’s columns [57]. Table 4 displays the MOA along with the column allocations. Error terms are stored in the MOA unassigned columns [58].

### 2.4. Response Variables

This study analyzes the corrosion resistance of the passivation coating of stainless steels using cyclic potentiodynamic polarization curves. Therefore, for this work, the corrosion current density (I_corr_) is taken as the response variable, which is obtained from the Tafel extrapolation method of the polarization curve [59]. A lower I_corr_ indicates that a given material has greater corrosion resistance [60].

### 2.5. Cyclic Potentiodynamic Polarization

CPPCs are performed using a potentiostat/galvanostat/ZRA (manufactured by Solartron 1287A, Bognor Regis, UK) in accordance with ASTM G61-86 [61], using a potential sweep from −0.80 to 1.60 V of the open circuit potential (OCP), a scan rate of 1 mV/s, and a full polarization cycle, the CPPC as performed. The passivated steels were evaluated in solutions of 1% *v*/*v* H_2_SO_4_ and 3.5% *w*/*v* NaCl at room temperature (25 ± 2 °C). Three electrodes make up the electrochemical cell. The working electrode, which is the sample under analysis, is formed by the passivated sample. The reference electrode is a saturated calomel electrode (SCE), which offers reliability for precisely measuring the applied potential. The counter electrode is a platinum electrode that creates a channel for the applied current to enter the solution. Because of the design of the cell, only 1 cm^2^ of the coated surface is exposed to the electrolyte. Before every experiment, the OCP is allowed to settle for 20 min. A computer records polarization data and controls the potentiostat. A customized tool for manually Tafel extrapolating the I_corr_ from the polarization graph is provided by a particular piece of software Cview 2. The Tafel extrapolation method consists of analyzing the linear regions of the anodic and cathodic branches of a potentiodynamic polarization curve, in the regions of approximately ±50 mV from the corrosion potential (E_corr_) and extrapolating them to their intersection to obtain the I_corr_. This methodology was used to calculate I_corr_ for all samples.

### 2.6. Taguchi Technique

By using orthogonal arrays (OAs) to minimize the number of tests needed, this work optimizes the experimental process using the Taguchi technique, a specific methodology within DOE [62]. When working with a lot of variables and limited resources, this approach works especially well. To ensure that every conceivable combination is investigated effectively, it arranges the experiments so that each row of an OA refers to a particular experiment and each column represents a factor with variable amounts [24]. The signal-to-noise ratio (S/N), which measures a process resistance to unpredictability, is a crucial part of the Taguchi method. There are the following two main scenarios for the S/N ratio: the “Smaller-the-better” scenario, which is used when reducing unwanted effects (such as corrosion rate or hardness in some studios), and the “Larger-the-Better” situation, which is used when the goal is to optimize the response (such as polarization resistance or tensile strength) [25]. Equation (1) is used to calculate the S/N ratio for the “Larger-the-Better” scenario. Equation (2) is used to calculate the S/N ratio for the “Smaller-the-Better” [25].

(1) $$S/N=-10\log\;\left(\frac{1}{n}\sum_{i=1}^{n}\frac{1}{y_{i}^{2}}\right)$$

(2) $$S/N=-10\log\;\left(\frac{1}{n}\sum_{i=1}^{n}y_{i}^{2}\right)$$

where *n* is the number of observations and *y_i_* is the observed data point, such as corrosion resistance in H_2_SO_4_ and NaCl solutions for a specific experiment. By methodically assessing the influence of several circumstances on the intended result, these equations guarantee the robustness and reliability of experimental data [63]. By combining these methodologies, the research aims to optimize the parameters of the passivation process that allow for the lowest corrosion current density in both solutions, which is reflected in a lower corrosion rate for PHSS.

#### 2.6.1. Analysis of Variance (ANOVA)

A crucial statistical technique for determining the relative impact of several processes factors and evaluating errors in experimentation is the analysis of variance (ANOVA). To better comprehend the relative influences on the output variables, ANOVA offers a quantified measure of each factor’s contribution. This method is crucial for detecting the disparity in prediction errors and any error variability in factor effects. Finding the design parameters that have a substantial impact on quality features is the main objective of an ANOVA. This analysis is usually carried out at a 5% significance level, which corresponds to a 95% confidence level [22,64].

#### 2.6.2. Percentage of Contribution in ANOVA

The percentage of contribution in an ANOVA shows how important each factor is in relation to the response variable [65]. It is calculated by Equation (3):

(3) $$\%\;\mathrm{Contribution}=\left(\frac{\mathrm{SS}\;\mathrm{Factor}}{\mathrm{SST}}\right)\times 100$$

where SS Factor is the sum of squares for each factor and SST is the total sum of squares for all factors. In the present case, ANOVA was used to analyze the percentage contribution of key input process parameters, such as the passivation bath solution (A), material (B), bath concentration (% *v*/*v*) (C), temperature (°C) (D), and time (min) (E) on the output variables of the PHSS passivation process.

This method offers a thorough comprehension of how each parameter affects PHSS corrosion resistance subjected to 1% *v*/*v* H_2_SO_4_ and 3.5% *w*/*v* NaCl solutions, facilitating the optimization of the passivation process for these AM350 and CUSTOM 450 alloys.

### 2.7. Gray Relation Analysis (GRA)

Gray relational analysis (GRA) estimates the interactions between multiple factors and responses, which helps in making comprehensive decisions [66]. GRA is a powerful instrument for optimizing multiple responses, commonly used in complex industrial processes such as machining, welding, plastic deformation, and wear, to name a few [67]. This approach is fundamental to our study on optimizing the PHSS passivation process, as it allows us to simultaneously evaluate corrosion resistance in two different solutions. This approach allowed for a more comprehensive decision-making process when determining the optimal conditions for the PHSS passivation process. By employing GRA, we made sure that the chosen parameters enhanced the corrosion resistance of the passivated materials in addition to improving individual attributes, which makes it extremely pertinent for aerospace applications.

GRA implies the linear normalization of experimental results, in this case I_corr_ in both test solutions in the range between 0 and 1. Normalization can be performed based on three objectives, including (1) normalization by maximum value (lower-the-better), (2) normalization by minimum value (higher-the-better), and (3) normalization by objective value [68].

#### 2.7.1. Normalization of Data

The first step in the GRA is data normalization, which scales the data within a range to allow comparison. The aim of this study is to increase the corrosion resistance of PHSS passivation coatings by achieving the minimum I_corr_ in the passivation coatings. Since a lower I_corr_ value indicates greater corrosion resistance, the normalization of I_corr_ is carried out using the lower-the-better criterion. The normalization expressions for the “higher-the-better” and “lower, the better” are presented in Equations (4) and (5); however, for this case only Equation (5) will be used.

(4) $$x_{i}\left(k\right)=\frac{y_{i}(k)-\min y_{i}(k)}{\max y_{i}\left(k\right)-\min y_{i}(k)}\;\;\;\;\;\;\;;\;(\mathrm{higher}\text{-}\mathrm{the}\text{-}\mathrm{better})$$

(5) $$x_{i}\left(k\right)=\frac{\max y_{i}(k)-y_{i}(k)}{\max y_{i}\left(k\right)-\min y_{i}(k)}\;\;\;\;\;\;\;;\;(\mathrm{lower}\text{-}\mathrm{the}\text{-}\mathrm{better})$$

In this case, *x_i_*(*k*) is the normalized value, max*y_i_*(*k*) are the maximum and min*y_i_*(*k*) are the minimum values of the *k*-th response. The response variable under discussion, in this case the I_corr_ in the H_2_SO_4_ and NaCl solution, is represented by the index *k*, whereas the index *i* relates to the experimental trial [68].

#### 2.7.2. Deviation of Data

After normalization, the deviation sequence is calculated to quantify the absolute difference between the normalized data and the reference sequence (ideal value). Equation (6) defines the deviation sequence ∆_0_*i*(*k*) [69]:

(6) $${∆}_{0}i=\left|x_{0}(k)-x_{i}(k)\right|$$

where ∆_0_*i*(*k*) is the deviation sequence for the *i*-th trial at the *k*-th response variable, *x*_0_(*k*) is the reference or ideal value for the *k*-th response, and *x_i_*(*k*) is the normalized value for the *i*-th trial and *k*-th response variable. The index *i* refers to the specific experimental trial, while *k* corresponds to the response variable being analyzed such as I_corr_ in H_2_SO_4_ and NaCl solutions. This deviation sequence provides a measure of how far each experimental run is from the ideal condition [69].

#### 2.7.3. Gray Relation Coefficient (GRC)

The association between reference and comparability sequences is measured by the GRC. GRC is evaluated using Equation (7) [41]:

(7) $$\xi_{i}\left(k\right)=\frac{{∆}_{\min}+r{∆}_{\max}}{{∆}_{0i}\left(k\right)+{r∆}_{\max}}$$

In Equation (7), *ξ_i_*(*k*) is the GRC for the *i*-th response at the *k*-th condition, ∆_min_ and ∆_max_ are the minimum and maximum deviation sequences, and *r* is the distinguishing coefficient, which is used to adjust the difference in the relational coefficient, typically set to 0.5 [41]. When Δ_max_ grows too large, the distinguishing coefficient reduces its impact and increases the relational coefficient’s distinct relevance. Because of the moderate distinguishing effects and strong result stability, the recommended value of the distinguishing coefficient, *r*, is 0.5. As a result, in this instance, *r*, is set at 0.5 for additional analysis [22].

#### 2.7.4. Gray Relational Grade (GRG)

The GRG provides an individual value, which represents the complete performance of each experimental condition. GRGs are estimated to express the relationship between the ideal (best = 1) and the actual experimental results. GRG is used in the GRA to clarify the connections between the series. The GRG, which is determined as follows, serves as the foundation for the overall evaluation of several response characteristics (Equation (8)) [22,41]

(8) $$\gamma_{i}=\frac{1}{n}\sum_{k=1}^{n}\xi_{i}(k)$$

where *γ_i_* is the GRG for the *i*-th experiment and *n* is the number of responses. The experimental result is closer to the ideal normalized value when the GRG is higher [22,41]. The appropriate parameter arrangement is therefore closer to the ideal when the gray relational grade is higher. By normalizing the data, determining the deviation sequences, and determining the GRC and GRG, we identify the optimal PHSS passivation parameters. This ensures robust passivation processes that produce superior corrosion resistance properties, improving the quality and reliability of the PHSS passivation coating.

## 3. Results

### 3.1. Cyclic Potentiodynamic Polarization Tests 

Table 5 displays the I_corr_ results found from the CPPC tests evaluated in a solution of 1% *v*/*v* H_2_SO_4_ y 3.5% *w*/*v* de NaCl. This study aims to obtain the lowest I_corr_ values, which are directly related to lower corrosion rates for PHSS passivated with the different parameters proposed in the DOE. The data in Table 5 will be used in the Taguchi method and in the GRA.

### 3.2. Taguchi Method

The Taguchi methodology converts the experimental data into a value for the evaluation characteristic in the optimal parameter analysis utilizing the S/N ratio. As shown in Table 6, for the H_2_SO_4_ solution the material is the parameter that has the highest influence on the I_corr_ and therefore on the corrosion rate, with a delta value of 8.3. This indicates that a variation in the material has a significant impact on the corrosion resistance of passivated PHSS. The passivating solution bath is the second most influential parameter in the passivation process, with a delta of 8.0, followed by the parameters of temperature, time, and solution concentration, with delta values of 5.4, 3.5, and 2.1, respectively. Each value in the table denotes the average S/N ratio at each parameter level, which helps reveal the optimal settings to minimize I_corr_ in 1% *v*/*v* H_2_SO_4_ solution. The delta value displays the difference between the highest and lowest S/N ratios for each parameter, where a higher delta reveals a greater influence on the response. The ranking column highlights the relative importance of each parameter, with the material being the most influential, followed by the passivating solution, temperature, time, and finally, the bath concentration. The “Smaller-the-Better” criterion was used to obtain the S/N ratio of I_corr_ in 1% *v*/*v* H_2_SO_4_ solution, as described in Equation (2).

Regarding I_corr_ in the 3.5% *w*/*v* NaCl solution (Table 6), the passivating solution was the most dominant parameter, with a delta of 10.2. The concentration of the passivating bath was the second largely influential, with a delta of 3.1, while time, temperature, and material had deltas of 2.5, 1.0, and 0.9, respectively, and were therefore fewer influential parameters. These delta results indicate the degree of variation in I_corr_ in the 3.5% *w*/*v* NaCl solution due to variations in each parameter. For this solution, the passivating solution had the greatest effect, followed by bath concentration, time, temperature, and material, as reflected in their ranking. As in the H_2_SO_4_ solution, the “Smaller-the-Better” criterion was applied, as defined in Equation (2), to calculate the S/N ratio values for I_corr_, since in this case the objective was to minimize I_corr_ and therefore the corrosion rate of the passivated PHSS. This highlights the need to carefully optimize the PHSS passivation parameters according to the specific requirements of the application, as different parameters apply varying degrees of influence on corrosion resistance in different test solutions.

**Figure 2:**
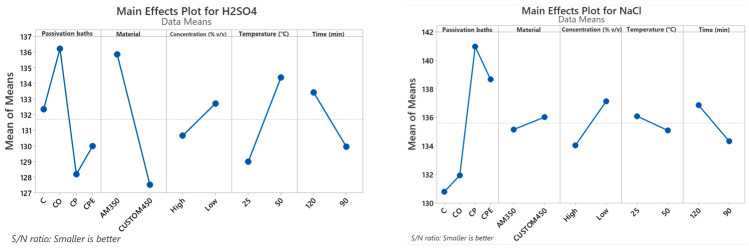
Taguchi analysis for I_corr_ in H_2_SO_4_ and NaCl solutions.

Figure 2 shows the S/N ratio for I_corr_ in H_2_SO_4_ and NaCl solution in relation to the following input passivation process parameters: passivation bath, material, concentration, temperature, and time. The response line deviation from the horizontal baseline shows how these parameters have a big influence on performance metrics. For I_corr_ in H_2_SO_4_ solution, the optimal levels were identified as a passivation bath of citric acid and hydrogen peroxide (CP) solution, CUSTOM 450, high concentration, temperature of 25 °C, and time of 90 min, in line with the “Smaller is better” criterion. For I_corr_ in NaCl solution, subsequent the same “Smaller is better” criterion, the following levels were determined to be optimal: passivation bath citric acid solution (C), AM350, high concentration, temperature 50 °C, and time 90 min. The results clearly indicate that the I_corr_ in both solutions is directly associated with the passivation states. In this sense, in the H_2_SO_4_ solution, the material and the passivation bath solution are the factors that have the greatest effect. In the case of I_corr_ in NaCl solution, the factors that significantly affect it are the passivation bath and the bath concentration. This is consistent with the findings of previous studies, which identified passivation bath and the bath concentration as critical parameters in passivation process [70,71]. The optimal balance of corrosion resistance is of utmost importance for aerospace components, which must withstand severe operating and maintenance conditions.

### 3.3. Gray Relation Analysis 

The multi-objective optimization problem, which is a passivation process, is better understood thanks to the GRA. The normalized values of I_corr_ obtained in a 1% *v*/*v* solution of H_2_SO_4_ and 3.5% *w*/*v* of NaCl solution were obtained using Equation (5) for both cases. For both test solutions, the criterion “smaller-the-better” was applied. Equation (6) was used to determine the deviation sequences. Next, the gray ratio coefficients (GRCs) were calculated with a distinctive coefficient *r* = 0.5 using Equation (7). Finally, the gray ratio degrees (GRGs) were estimated using Equation (8), which represents the general performance of the PHSS passivation process parameters.

**Figure 3:**
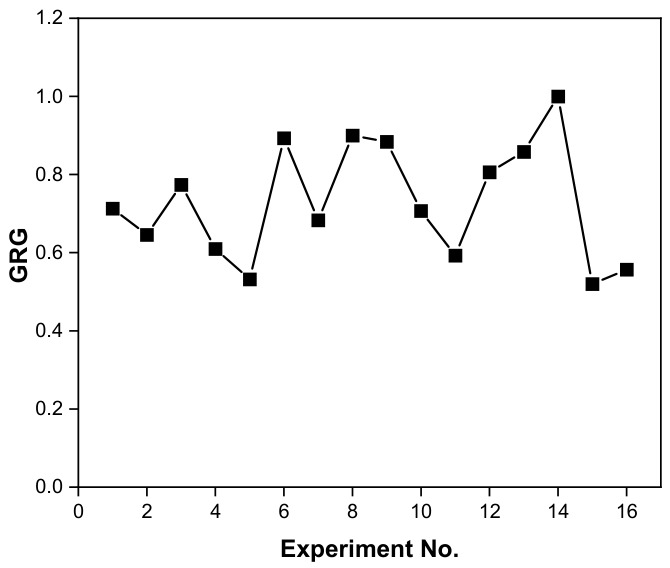
GRG/experiment.

The appropriate mix of elements is generally closer to the ideal state when the GRG is higher. The parameter values for experiment No. 14 produced the highest GRG, as seen in Table 7 and Figure 3. As a result, out of the sixteen experiments, experiment No. 14 has the greatest multiple performance characteristics. Thus, the combined Taguchi and gray relation analysis approach was used to convert this multi-criteria optimization problem into a single-objective optimization problem. Additionally, the “Larger-the-Better” criterion was used to determine the S/N ratio for the overall gray relation degree, as shown in Equation (1).

**Figure 4:**
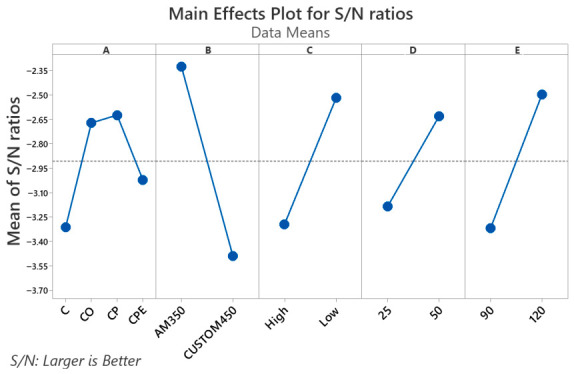
S/N ratio plot for the overall GRG.

The S/N ratio for the GRG rating is shown visually in Figure 4, where the dashed line shows the overall mean value of the S/N ratio. Figure 4 shows the main effects plot for GRG. The principal effect plot provides the optimal combination of passivation process parameters to achieve maximum corrosion resistance. The higher the degree of gray relationship, the closer the product quality will be to the ideal value. Therefore, the optimal combination of passivation process parameters to minimize I_corr_ in H_2_SO_4_ and NaCl solutions, presenting less variability at these levels, is A3B1C1D2E2. The principal effect plot also provides a rough idea of the relative importance of the parameters in the system response. If the plot for a particular parameter has the steepest slope, then that parameter is the most important. Conversely, a plot that is close to horizontal is not important. Figure 4 shows that parameter B is the highest important, followed by parameters E, C, A, and D, respectively. A greater GRG denotes a stronger correlation since it gauges the association between reference and comparability sequences. The GRG rating for each control factor level is indicated in the response table (Table 8).

The integration of the Taguchi technique with GRA allowed for comprehensive optimization of PHSS passivation parameters. The optimal settings identified were as follows: the passivation bath, a mixture of citric acid and oxalic acid, AM350 material, high concentration of the bath, temperature 50 °C, and time 120 min, significantly improving corrosion resistance properties by decreasing I_corr_, which is essential for aerospace applications. This method provides a solid framework for systematically evaluating and improving the PHSS passivation process, combination Taguchi’s design of experiments with GRA, presenting a comprehensive approach to multi-objective optimization in the passivation process. The combined approach of the Taguchi methodology and GRA offers greater reliability, experimental efficiency, and practical applicability, making it particularly well-suited for optimization studies in materials engineering, corrosion, tribology, and advanced manufacturing processes.

### 3.4. ANOVA

The research results of the analysis of variance (ANOVA) performed to determine the important control factors influencing performance attributes are shown in this section, specifically corrosion resistance, by obtaining the lowest I_corr_ in solutions of 1% *v*/*v* H_2_SO_4_ and 3.5% *w*/*v* NaCl. The results of the ANOVA for I_corr_ in 1% *v*/*v* H_2_SO_4_ and 3.5% *w*/*v* NaCl solutions are presented in Table 9. A high F-value shows a significant effect of the parameter on the performance characteristic.

The material was revealed to be the highest significant parameter, with a contribution of 32.73%, suggesting its dominant influence on corrosion resistance with a low I_corr_. The passivation bath and temperature contributed 16.98% and 13.70%, respectively. Meanwhile, time and concentration bath contributed 5.69% and 2.01% each; therefore, their contribution to the passivation process is not as significant. These results indicate that variations in material, passivation bath, and temperature significantly affect I_corr_ when evaluated in H_2_SO_4_ solution, while bath time and concentration have minor but noticeable effects.

The ANOVA results for I_corr_ in a 3.5% *w*/*v* NaCl solution reveal that the passivation bath is the highest influential parameter, with a contribution of 37.39%. This high percentage highlights its important position in determining I_corr_ in a NaCl solution. This is followed by bath concentration, with a contribution of 4.80%, and time, with a contribution of 3.21%. Temperature and material are the least influential factors, with contributions of 0.50% and 0.37%, respectively. Influential contributions of each input parameter and verifying the outcomes of the Taguchi method required the use of the ANOVA approach.

The ANOVA results for the overall gray relationship degree of the response to corrosion resistance using I_corr_ were obtained using Minitab Statistical Software, version 21 [58] and are shown in Table 10. Analysis of variance is used to obtain a quantified idea of the effect of the design parameters (A, B, C, D, and E) and their interactions (A × B, A × C, A × D, A × E, B × C, B × D, B × E, etc.) on the polarization characteristics of the passivation coating in PHSS. The Taguchi experimental method was unable to evaluate the effect of separate parameters throughout the process, so the percentage contribution using ANOVA is used to compensate for this effect. The ANOVA table also includes F-values. By comparation the evaluated F-values with the tabulated ones, the importance of the factors and their interactions can be easily understood. If the F-value obtained from a parameter or interaction is greater than the tabulated one, then that particular parameter or interaction has a significant influence on the process response. Table 10 shows that the interaction of parameters A × B, i.e., the passivating bath and the material, has the most significant influence on the polarization characteristics, with 38.81%; the interaction of parameters A × E (passivating bath and time) has a large effect on the passivation process, with a contribution of 25.74%. In the case of individual parameters, material (B) is the parameter that contributes most to the passivation characteristics, with 11.75%, followed by parameters E, C, D, and A with contribution percentages of 5.87%, 5.51%, 3.76%, and 3.31%, respectively. The B × E interaction contributes only 0.03% and can therefore be considered insignificant in the PHSS passivation process.

### 3.5. Confirmation Test

The last stage next determining the ideal stage combination of the design parameters is to confirmation test any improvement in the outcomes truly takes place at the optimal condition as opposed to the original condition. The ideal levels of the control parameters are used in this test. Equation (9) [22,41] can be used to predict the signal-to-noise (S/N) ratio and the GRG:

(9) $$\eta_{opt}=n_{m}+\sum_{i=j}^{n}\left(n_{j}-n_{m}\right)$$

where *n_m_* represents the overall mean of the S/N or GRG, *n_j_* represents the mean S/N or GRG at the optimized level, and *n* denotes the number of significant passivation process parameters. Table 11 shows the gray ratio at the initial condition, the experimental gray ratio, and the predicted gray ratio. In this study, a combination of medium GRG levels for the passivation coating parameters is supposed as the initial condition since, as mentioned above, experiment No. 14 has a GRG of 1.00 and cannot be taken as the initial condition. This is because, as observed in the ANOVA of the GRG data, there are strong interactions between the passivation bath parameter (A) and the material (B) and time (E) parameters. From the table, it can be seen that the enhancement in the gray ratio at the optimal condition is 0.340, which is approximately 55.73% of the average gray ratio. This is seen as a major advancement. Figure 4 shows the polarization curves of the coatings created under both initial and ideal conditions. As expected, the polarization curves showed that the experimentally optimized passivated coatings display better corrosion resistance than the initial condition and the optimal condition of experiment 14, due to the interactions of factor A with other factors such as factors B and E.

These findings emphasize the significance of a confirmation test to verify the ideal parameters found in the preliminary study. The consistency and efficacy of the statistical optimization approach are validated by determining the ideal S/N ratio and GRG and confirming these predictions with real experimental data. The significant improvements in corrosion resistance demonstrated by lower I_corr_ values in H_2_SO_4_ and NaCl solutions validate the practical advantages of this procedure for improving the performance of the PHSS passivation process, making it highly relevant for aerospace applications where corrosion resistance under different conditions is critical for PHSS [72].

**Figure 5:**
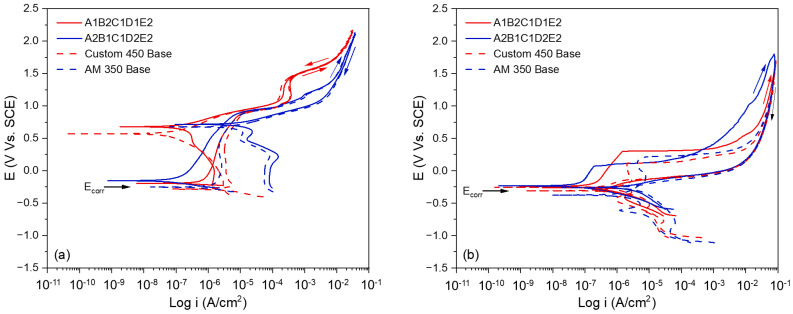
Cyclic potentiodynamic polarization curves for passivation coatings evaluated in (**a**) H_2_SO_4_ solution and (**b**) NaCl solution.

The potentiodynamic polarization curves obtained in H_2_SO_4_ show (Figure 5a), in general terms, well-defined passivation regions for all the samples evaluated, characterized by an active–passive transition at positive potentials (E_A–C_), with values ranging between 0.571 V and 0.715 V vs. SCE. This response indicates the formation of stable passive films in the acidic medium, which is consistent with the typical behavior of stainless steels in H_2_SO_4_ solutions.

From a thermodynamic point of view, Table 12 shows that sample A2B1C1D2E2 exhibits the most noble corrosion potential (E_corr_ = −0.155 V vs. SCE), indicating a lower spontaneous tendency to corrode in this medium. In contrast, CUSTOM 450 has the most active E_corr_ (−0.284 V vs. SCE), suggesting greater thermodynamic instability at the metal-solution interface. Samples A1B2C1D1E2 (−0.195 V vs. SCE) and AM 350 (−0.253 V vs. SCE) are located in intermediate positions within this nobility scale. In terms of corrosion kinetics, the corrosion current density (I_corr_) reveals substantial differences between the materials. A2B1C1D2E2 has the lowest I_corr_ in the group (5.646 × 10^−8^ A/cm^2^), which translates into the lowest uniform corrosion rate in H_2_SO_4_. A1B2C1D1E2 occupies a favorable intermediate position (5.364 × 10^−7^ A/cm^2^), while CUSTOM 450 Base has the highest I_corr_ (3.623 × 10^−6^ A/cm^2^), approximately 64 times higher than that of A2B1C1D2E2, which shows a considerably higher dissolution rate under these conditions.

Analysis of the return scan hysteresis allows us to distinguish between the dominant corrosion mechanisms. Samples A2B1C1D2E2 and AM 350 Base exhibit positive hysteresis, indicating the development of stable pitting corrosion with relatively high initiation potentials E_pit_ = 0.865 V and 0.869 V vs. SCE, respectively. These E_pit_ values suggest that, although both samples are susceptible to pitting, this only occurs at very high anodic potentials, offering considerable protection under normal service conditions. On the other hand, A1B2C1D1E2 and CUSTOM 450 Base exhibit negative hysteresis, indicating that the material repassivates before pitting stabilizes during the return scan. This behavior is characteristic of materials with good repassivation capacity in H_2_SO_4_, in which corrosion tends to be uniform rather than localized.

The electrochemical behavior in NaCl (Figure 5b) differs significantly from that observed in H_2_SO_4_, reflecting the highly aggressive nature of the chloride ion (Cl^−^) on the passive films of stainless steels. The most relevant feature is that all samples exhibit positive hysteresis in this medium, indicating that Cl^−^ universally promotes the initiation and stabilization of pitting corrosion, regardless of the alloy composition.

Table 13 shows that the active-passive potentials (E_A–C_) are negative for all samples in NaCl (between −0.236 V and −0.261 V vs. SCE), in contrast to the positive values recorded in H_2_SO_4_. This cathodic shift reflects the preferential adsorption of Cl^−^ in passive film defects, which weakens protection and facilitates the transition to the active state at lower potentials. This fundamental difference explains the greater susceptibility to localized corrosion observed in this medium. Pitting potentials in NaCl are considerably lower than in H_2_SO_4_ for all samples, ranging between 0.071 V vs. SCE (A2B1C1D2E2) and 0.298 V vs. SCE (A1B2C1D1E2). These reduced values imply that pitting initiation occurs at more accessible potentials under service conditions, increasing the risk of localized corrosion.

In terms of corrosion potential, the values in NaCl are significantly more negative than in H_2_SO_4_ for all samples, with AM 350 exhibiting the most active E_corr_ (−0.376 V vs. SCE). A1B2C1D1E2 and A2B1C1D2E2 exhibit similar values −0.244 V vs. SCE and −0.234 V vs. SCE, respectively, while CUSTOM 450 stands at −0.308 V vs. SCE. This general shift towards more active potentials reflects the interaction of Cl^−^ with the metal surface and the weakening of the thermodynamic stability of the passive layer. The corrosion current density in NaCl shows a significant redistribution compared to sulfuric acid. A2B1C1D2E2 remains the sample with the highest kinetic resistance, having the lowest I_corr_ value in the group (7.859 × 10^−8^ A/cm^2^), the same position it held in H_2_SO_4_. The latter undergoes a somewhat more drastic degradation upon changing media; its I_corr_ increases from 5.646 × 10^−8^ to 7.859 × 10^−8^ A/cm^2^, an increase of approximately 1.4 times, suggesting that its composition, which is favorable for passivation in H_2_SO_4_, is particularly vulnerable to the destabilizing action of Cl^−^.

## 4. Discussion

### 4.1. Taguchi Method

A notable finding of Taguchi’s analysis is the hierarchy of influence of the parameters depending on the evaluation solution. In the 1% *v*/*v* H_2_SO_4_ solution, the material (B) was found to be the highest influential parameter, with a delta value of 8.3, closely followed by the passivating bath solution (A) with a delta of 8.0. Temperature (D), time (E), and bath concentration (C) had progressively lower delta values of 5.4, 3.5, and 2.1, respectively. This hierarchy indicates that the intrinsic electrochemical properties of the alloy, particularly its chromium content, microstructural constitution, and degree of precipitation hardening, play a dominant role in corrosion resistance when evaluated in a sulfated acidic medium. The narrow margin between parameters A and B (delta difference in only 0.3) further suggests a strong coupling between the composition of the material and the nature of the passivating agent, an observation later confirmed by ANOVA interaction analysis. In contrast, for I_corr_ in the 3.5% *w*/*v* NaCl solution, the passivation bath (A) became the greatest influential parameter with a delta of 10.2, while the material (B) dropped to the least influential position with a delta of only 0.9. This reversal in the parameter hierarchy is particularly significant and reflects the fundamentally different mechanism by which chloride ions attack the passive film compared to sulfate ions. In NaCl media, the selective adsorption of Cl^−^ on defects in the passive film compromises the integrity of the chromium oxide layer regardless of the base alloy, making the chemical composition and oxidizing capacity of the passivation bath the determining factor for protection. As a result, bath concentration (C) emerged as the second highest influential parameter (delta = 3.1), followed by time (E), with delta of 2.5, temperature (D), with delta of 1.0, and material (B), with delta of 0.9.

This solution-dependent change in parameter dominance closely resembles observations reported in the literature on manufacturing process optimization. Philip Selvaraj et al. [73] reported an analogous phenomenon in the dry turning of nitrogen-alloyed duplex stainless steel, where feed rate was the main parameter for surface roughness, but cutting speed governed tool wear, two responses that react to the same process through fundamentally different physical mechanisms. Similarly, the PHSS passivation process exhibits dual sensitivity: the alloy governs resistance to uniform dissolution in H_2_SO_4_, while the chemistry of the passivating bath determines resistance to localized attack (pitting) in NaCl. Hascalik and Caydas [74] similarly identified that feed and cutting speed specialize in controlling surface roughness and tool life, respectively, in the turning of Ti-6Al-4V, demonstrating that parameter specialization according to response type is a recurring pattern in the optimization of surface-related processes in high-performance engineering materials.

The optimal levels identified by analyzing the S/N ratio also reflect this dual response behavior. For I_corr_ in H_2_SO_4_ solution, the optimal combination consisted of citric acid + hydrogen peroxide (CP) bath, CUSTOM 450 alloy, high concentration, 25 °C, and 90 min. In contrast, for I_corr_ in NaCl solution, the optimal levels shifted towards citric acid bath (C), AM350 alloy, high concentration, 50 °C, and 90 min. The divergence in the optimal material and bath solution between the two environments is consistent with the electrochemical results discussed in Section 3.5, where sample A2B1C1D2E2 exhibited superior performance in H_2_SO_4_ but was particularly vulnerable in NaCl due to its sensitivity to Cl^−^-induced passive film destabilization. This conflict between the individual optima of each solution provides the fundamental justification for employing GRA as a multi-objective integration tool [75].

From a practical perspective, Taguchi’s analysis demonstrated that the mixed orthogonal array L16 (4^1^ 2^4^) provided an efficient experimental framework that reduced the experimental burden of the 64 trials required by a full factorial design to only 16 runs, while preserving orthogonality and balance among all factor levels. This gain in efficiency, widely documented in the literature [19,22], is particularly valuable for the development of the passivation process in the aeronautical context, where material costs and testing infrastructure impose significant constraints on the experimental volume.

### 4.2. Gray Relation Analysis 

The methodological sequence followed in this work is consistent with the practice established in the literature on GRA applied to electrochemical and surface treatment processes. Kao and Hocheng [76] demonstrated the efficiency of this procedure in optimizing the electropolishing of 316L stainless steel, applying the same min–max normalization scheme, distinguishing coefficient *r* = 0.5, and GRG calculation as the average of the gray ratio coefficients to consolidate responses with conflicting criteria, surface roughness “lower-is-better” and passivation resistance “higher-is-better”. In this work, the normalization of I_corr_ in both solutions was performed under the “lower-is-better” criterion using Equation (5), since a lower I_corr_ value directly represents greater corrosion resistance of the passive coating.

The GRA results identified experiment No. 14, corresponding to combination A3B1C1D2E2 (citric acid + oxalic acid bath, AM350, concentration 25% *v*/*v*, temperature 50 °C, time 120 min), as the one with the highest GRA with a value of 1.00, indicating that this experimental condition achieved the behavior closest to the ideal reference considering simultaneously the corrosion resistance in H_2_SO_4_ and NaCl. This result is coherent with the interpretative logic proposed by Cao et al. [77], who established that a higher GRA denotes a closer correlation with the ideal reference behavior, and whose interpretative scale (where values above 0.9 indicate significant influence) places experiment No. 14 in the optimal performance range. The analysis of the main effects of the GRA (Figure 4 and Table 8) revealed that parameter material (B) was the individual factor with the highest slope in the main effects graph, positioning itself as the greatest influential on the overall performance of the passivation process, followed by parameter time (E), concentration (C), passivating bath (A), and temperature (D). This hierarchy, obtained using the “higher-is-better” criterion on the GRA (Equation (1)), differs significantly from the individual hierarchies determined in the Taguchi analysis for each solution separately, where the passivating bath and the material alternated between each other in the dominant positions. The convergence of the integrated analysis around the material as the dominant factor suggests that, when considering performance in both environments in a weighted manner, the intrinsic electrochemical stability of the alloy and its ability to form and maintain a resistant passive film in both sulphated acidic and chloride environments is the property that most influences the overall result of the treatment. This observation coincides with Das and Sahoo findings [78], who applied the Taguchi-GRA methodology to the optimization of Ni-B electrolytic coatings deposited on AISI 1040 steel, simultaneously evaluating the charge transfer resistance (R_ct_) and the double layer capacitance (C_dl_) obtained by Electrochemical Impedance Spectroscopy (EIS). In that work, the deposition bath temperature emerged as the most influential parameter on the combined GRA (delta = 0.1962), despite the fact that its influence on each individual response was different, similar to what was observed in the present study, where the material dominates the integrated GRA even though its contribution in NaCl is practically negligible (0.37% according to ANOVA). This behavior shows that multi-response integration via GRA can redistribute the hierarchy of importance of parameters with respect to univariate analysis, especially when individual responses respond asymmetrically to the same factor.

The optimal combination identified by the GRA (citric acid bath + oxalic acid (A2), AM350 (B1), high concentration (C1), temperature of 50 °C (D2), and time of 120 min (E2)) can be interpreted from a consistent electrochemical perspective. The mixed bath of citric acid and oxalic acid combines the chelating capacity of citrate for the removal of free iron with the degreasing and activating action of oxalate, promoting the formation of a dense and uniform passive layer enriched in chromium on the surface of the PHSS [11,12]. The temperature of 50 °C and the time of 120 min enhance the kinetics of this layer’s formation without compromising the integrity of the substrate, while the concentration of 25% *v*/*v* ensures sufficient chemical activity for the treatment of larger surface areas. The selection of AM350 as the optimal material factor level is consistent with the electrochemical results, where this alloy showed less susceptibility to destabilization of the passive film in NaCl compared to CUSTOM 450, a behavior attributable to its higher relative chromium content and microstructure.

Overall, the GRA results demonstrate that the Taguchi-GRA method provides a robust and effective framework for the multi-objective optimization of the PHSS passivation process, capable of resolving the conflict between the optimal conditions in H_2_SO_4_ and NaCl by constructing a global performance index.

### 4.3. ANOVA Methodologies

The ANOVA results provide a statistically rigorous quantification of the contribution of separate passivation parameter to the observed variability in corrosion current density, complementing and validating the S/N relationship hierarchies found using the Taguchi method. For I_corr_ in the 1% *v*/*v* H_2_SO_4_ solution, material (B) accounted for the largest fraction of total variability, with a contribution of 32.73%. This finding confirms the dominant role of alloy composition and microstructural constitution in generalized corrosion resistance in an acidic environment. The passivating bath (A) and temperature (D) contributed 16.98% and 13.70%, respectively, indicating that, although the chemical nature of the passivating agent significantly modifies the structure of the passive layer, the thermal activation of film formation reactions also plays a relevant role. Time (E) and bath concentration (C) exhibited substantially smaller contributions of 5.69% and 2.01%, indicating that, within the ranges studied, prolonging the immersion time or varying the concentration produces marginal improvements in resistance in H_2_SO_4_ once the thermodynamically stable passive film has been established. In contrast, in the 3.5% *w*/*v* NaCl solution, the contribution pattern was markedly different. The passivating bath (A) dominated with 37.39%, reaffirming the critical role of bath chemistry in determining chloride-induced pitting resistance. The remaining parameters contributed considerably less: bath concentration (C) with 4.80%, time (E) with 3.21%, temperature (D) with 0.50%, and material (B) with only 0.37%. The virtually zero contribution of material in NaCl (0.37% versus 32.73% in H_2_SO_4_) is particularly revealing, as it indicates that the protective capacity conferred by the passivation treatment greatly outweighs the inherent differences between AM350 and CUSTOM 450 in chloride environments. The key differentiation between the samples in NaCl is therefore determined by the film formed during passivation and not by the composition of the substrate.

These contribution patterns are consistent with similar findings reported in the literature on manufacturing process optimization. Philip Selvaraj et al. [73] reported that feed contributed between 63 and 64% to the variability of surface roughness in the turning of duplex stainless steel, while cutting speed accounted for between 91 and 92% of tool wear, a clear demonstration of the dominance of response-specific parameters, analogous to the material/bath dichotomy observed in the present work. Similarly, Hascalik and Caydas [74] found that feed dominated surface roughness (54.5%) while cutting speed dominated tool wear (73.7%) in the turning of Ti-6Al-4V, reinforcing the principle that in complex multi-response systems, a single parameter rarely governs all responses simultaneously. The ANOVA results presented in this work extend this principle to surface treatment processes, demonstrating that passivating bath chemistry and alloy selection operate through mechanistically distinct pathways depending on the corrosive environment.

The ANOVA of the gray relationship analysis (GRA) introduced an additional dimension of analysis by quantifying both the individual contributions of the parameters and their interaction effects on the integrated multi-response performance index. In this analysis, material (B) remained the most influential individual parameter with a contribution of 11.75%, followed by time (E), with 5.87%, bath concentration (C), with 5.51%, temperature (D) with 3.76%, and passivating bath (A) with 3.31%. However, the most relevant finding was the dominant contribution of interaction effects: the A × B interaction (passivating bath × material) explained 38.81% of the total variability, and the A × E interaction (passivating bath × time) contributed an additional 25.74%. Together, these two interactions explain approximately 64.5% of the total variance in GRA, far exceeding the combined contribution of all individual factors (30.2%). The predominance of interaction effects over main effects in the ANOVA of the GRG is a highly relevant finding with important implications for process design and control. It indicates that the effectiveness of a given passivation bath solution is not a fixed property but rather depends heavily on the alloy being treated and the immersion time applied. For example, a bath composition that produces excellent results on AM350 may not offer comparable performance on CUSTOM 450, and vice vers. This contextual dependence would be completely invisible in an analysis limited to individual response S/N ratios or in a lower-resolution experimental design incapable of detecting interaction effects. The dominance of the A × B interaction is analogous to the multifactor coupling observed by Kumar et al. [79] in TIG welding of AISI 304, where voltage and root separation exhibited significant interaction effects that shifted the optimal combination of parameters depending on whether hardness or bending strength was the target response.

Finally, the ANOVA results reinforce the conclusion that optimizing the PHSS passivation process for aerospace applications requires a multi-solution, multi-response approach. The divergent contribution hierarchies between the H_2_SO_4_ and NaCl environments, the strong interaction effects revealed in the GRA, and the alloy-specific sensitivities collectively demonstrate that a single-parameter, single-response optimization strategy would be fundamentally insufficient for this process.

### 4.4. Confirmation Test

The quantified improvement in GRA under optimal conditions compared to the initial condition was 0.340, equivalent to 55.73% of the average relational degree. This result robustly validates the effectiveness of the combined methodology, falling within a range of improvement consistent with that reported in similar studies in literature. Kao and Hocheng [76] obtained a relational degree improvement of 0.3338 in the optimization of 316L stainless steel electropolishing using GRA with an L9 arrangement, experimentally verifying that the optimal parameters reduced surface roughness from 50% to 4.75% and increased passivation resistance to 100%. Das and Sahoo [78] reported a GRA improvement of 0.3239 in the optimization of Ni-B coatings characterized by EIS, equivalent to 53% of the average relational degree. The convergence of the improvement percentages among the three studies, despite differences in material, process, and number of experiments, reinforces the consistency and robustness of the Taguchi-GRA methodology as a tool for optimizing surface treatments in metal systems with multiple corrosion responses.

Analysis of CPPC obtained under optimal conditions, initial conditions, and experiment No. 14 in 1% *v*/*v* H_2_SO_4_ solution reveals significant electrochemical differences that support the predictions of the statistical analysis. Sample A2B1C1D2E2 exhibited the most noble corrosion potential of the group (E_corr_ = −0.155 V vs. SCE), a minimum I_corr_ of 5.646 × 10^−8^ A/cm^2^, and a pitting potential of 0.865 V vs. SCE, with positive hysteresis indicating the formation of stable pits only at very high anodic potentials. This combination of electrochemical parameters reflects a passive film of high integrity and surface density, consistent with the interactive action of the citric acid + oxalic acid bath on the surface of AM350 at 50 °C for 120 min. The behavior in a 3.5% *w*/*v* NaCl solution introduces an additional layer of complexity that highlights the importance of the multi-objective approach adopted in this study. In this medium, sample A2B1C1D2E2 exhibited the lowest I_corr_ of the group (7.859 × 10^−8^ A/cm^2^), as well as the lowest pitting potential (E_pit_ = 0.071 V vs. SCE). Even more revealing, sample A2B1C1D2E2 underwent drastic degradation upon changing media: its I_corr_ increased from 5.646 × 10^−8^ to 7.859 × 10^−8^ A/cm^2^, an increase of approximately 1.4 times, attributable to the particular susceptibility of the conformation of the passive film formed in this bath to competitive adsorption of Cl^−^ at the active sites on the surface. This differential behavior between solutions is precisely the experimental manifestation of the optimization conflict detected in Taguchi’s analysis and constitutes the most solid electrochemical justification for the use of GRA without multi-objective integration. An optimization based exclusively on H_2_SO_4_ would have selected conditions that are particularly vulnerable in actual service, where PHSS aeronautical components are simultaneously exposed to acidic media during maintenance procedures and to chloride environments during operation.

The set of confirmation test results acquires its fullest meaning when interpreted in the context of the service requirements of PHSS aeronautical components. The AM350 and CUSTOM 450 alloys are used in landing gear systems, structural fasteners, and engine components that alternate between exposure to acidic maintenance fluids and operating environments with varying concentrations of chlorides from seawater, deicing salts, and coastal atmospheric pollutants during their service life [1,2,4]. The Taguchi-GRA methodology applied in this work ensures that the optimized passivation conditions offer a robust compromise between both environments, exceeding the performance of the non-optimized conditions by 55.73% when both responses are weighted in an integrated manner. This level of improvement, validated experimentally and consistent with the specialized literature [35,76,80,81,82], supports the practical viability of citric acid + oxalic acid solutions as a nitric acid-free alternative for PHSS passivation in the aeronautical industry, in line with current trends toward more sustainable surface treatment processes and in compliance with emerging environmental regulations on the use of strong oxidizing agents in aircraft maintenance facilities.

## 5. Conclusions

- The parameters of the passivation coating process, such as passivation bath, material, bath concentration, temperature, and time, are optimized to maximize corrosion resistance by obtaining the lowest I_corr_ values in HPSS. This multiple response problem is well optimized by using Taguchi design of experiments in conjunction with GRA.
- The optimal combination of parameters has been determined to be A2B1C1D2E2 (passivation bath solution formed by a mixture of citric acid and oxalic acid, HPSS AM350, low concentration level, high temperature level (50 °C), and high time level (120 min)).
- According to the ANOVA method, the material significantly influences corrosion resistance in H_2_SO_4_ solution, with a contribution of 32.73%, while the passivation bath is the factor that most influences corrosion resistance in NaCl solution, with a contribution of 37.39%.
- The ANOVA results for the GRG data indicated that the material (11.75%) was the factor that most influenced the achievement of optimal passivation coating results, followed by time (5.87%), bath concentration (5.51%), temperature (3.37%), and finally, the passivation bath (3.31%). It was observed that the interactions between the passivation bath and material contribute 38.81%, and the interaction between the passivation bath and time contributes 25.74% to the HPSS passivation process.
- The application of statistical optimization techniques (Taguchi–GRG–ANOVA) resulted in a significant enhancement of HPSS corrosion resistance, with an increase of about 55% compared to the untreated state.

## Figures and Tables

**Table 1:** Chemical composition of AM 350 and CUSTOM 450 precipitation-hardening stainless steel (wt.%).

Material	Elements (wt.%)										
	Fe	Cr	Ni	C	Mo	Mn	Si	Cu	Ti	Nb	S
AM 350	Bal.	16.0–17.0	4.0–5.0	0.07–0.11	2.50–3.25	0.50–1.25	≤0.50	-	-	-	0.030
CUSTOM 450	Bal.	14.0–16.0	5.0–7.0	≤0.05	0.50–1.0	1.00	1.00	1.25–1.75	0.90–1.40	0.5–0.75	0.030

**Table 2:** Parameters to passivation treatment.

Parameters	Passivation Process
Passivation baths	(a) citric acid (c) (b) citric acid and oxalic acid (co) (c) citric acid and hydrogen peroxide (cp) (d) citric acid, hydrogen peroxide, and ethanol (cpe)
Material	AM 350, CUSTOM 450
Concentration (% *v*/*v*)	Low, high
Temperature (°C)	25, 50
Time (min)	90, 120

**Table 3:** Design parameters and levels.

Parameters	Units	Level 1	Level 2	Level 3	Level 4
Passivation baths (A)		(a) Citric acid	(b) Citric acid and oxalic acid	(c) Citric acid and hydrogen peroxide	(d) Citric acid, hydrogen peroxide, and ethanol
Material (B)		AM 350	CUSTOM 450		
Concentration (C)	(% *v*/*v*)	Low	High		
Temperature (D)	(°C)	25	50		
Time (E)	(min)	90	120		

**Table 4:** Taguchi’s L16 mixed orthogonal array (4^1^, 2^4^).

Number	Input Parameters				
	A	B	C	D	E
1	Citric acid	AM 350	Low	25	90
2	Citric acid	AM 350	High	50	120
3	Citric acid	CUSTOM 450	High	50	90
4	Citric acid	CUSTOM 450	Low	25	120
5	Citric acid and oxalic acid	AM 350	High	50	90
6	Citric acid and oxalic acid	AM 350	Low	25	120
7	Citric acid and oxalic acid	CUSTOM 450	Low	25	90
8	Citric acid and oxalic acid	CUSTOM 450	High	50	120
9	Citric acid and hydrogen peroxide	AM 350	Low	50	90
10	Citric acid and hydrogen peroxide	AM 350	High	25	120
11	Citric acid and hydrogen peroxide	CUSTOM 450	High	25	90
12	Citric acid and hydrogen peroxide	CUSTOM 450	Low	50	120
13	Citric acid, hydrogen peroxide, and ethanol	AM 350	High	25	90
14	Citric acid, hydrogen peroxide, and ethanol	AM 350	Low	50	120
15	Citric acid, hydrogen peroxide, and ethanol	CUSTOM 450	Low	50	90
16	Citric acid, hydrogen peroxide, and ethanol	CUSTOM 450	High	25	120

**Table 5:** I_corr_ obtained of CPPC tests.

S. No.	Uncoded Matrix					Average Results	
	A	B	C	D	E	I_corr_ (A/cm^2^) H_2_SO_4_	I_corr_ (A/cm^2^) NaCl
1	Citric acid	AM 350	Low	25	90	2.364 × 10^−7^	2.505 × 10^−7^
2	Citric acid	AM 350	High	50	120	1.208 × 10^−7^	7.591 × 10^−7^
3	Citric acid	CUSTOM 450	High	50	90	2.211 × 10^−7^	1.623 × 10^−7^
4	Citric acid	CUSTOM 450	Low	25	120	5.364 × 10^−7^	2.246 × 10^−7^
5	Citric acid and oxalic acid	AM 350	High	50	90	2.238 × 10^−7^	1.032 × 10^−6^
6	Citric acid and oxalic acid	AM 350	Low	25	120	7.215 × 10^−8^	1.665 × 10^−7^
7	Citric acid and oxalic acid	CUSTOM 450	Low	25	90	3.272 × 10^−7^	2.212 × 10^−7^
8	Citric acid and oxalic acid	CUSTOM 450	High	50	120	1.084 × 10^−7^	1.078 × 10^−7^
9	Citric acid and hydrogen peroxide	AM 350	Low	50	90	1.451 × 10^−7^	8.878 × 10^−8^
10	Citric acid and hydrogen peroxide	AM 350	High	25	120	5.224 × 10^−7^	9.001 × 10^−8^
11	Citric acid and hydrogen peroxide	CUSTOM 450	High	25	90	9.418 × 10^−7^	1.356 × 10^−7^
12	Citric acid and hydrogen peroxide	CUSTOM 450	Low	50	120	3.223 × 10^−7^	5.921 × 10^−8^
13	Citric acid, hydrogen peroxide, and ethanol	AM 350	High	25	90	2.114 × 10^−7^	6.572 × 10^−8^
14	Citric acid, hydrogen peroxide, and ethanol	AM 350	Low	50	120	6.177 × 10^−8^	5.041 × 10^−8^
15	Citric acid, hydrogen peroxide, and ethanol	CUSTOM 450	Low	50	90	9.439 × 10^−7^	2.537 × 10^−7^
16	Citric acid, hydrogen peroxide, and ethanol	CUSTOM 450	High	25	120	8.103 × 10^−7^	2.198 × 10^−7^

**Table 6:** S/N ratio response for 1% *v*/*v* H_2_SO_4_ and 3.5% *w*/*v* NaCl solution.

		**Level**	**Passivation** **Baths**	**Material**	**Concentration**	**Temperature** **°C**	**Time** **min**
Smaller-the-Better	1% *v*/*v* H_2_SO_4_	1	132.4	135.9	130.7	129	133.4
		2	136.2	127.5	132.7	134.4	130
		3	128.2				
		4	130				
		Delta	8	8.3	2.1	5.4	3.5
		Rank	2	1	5	3	4
	3.5% *w*/*v* NaCl	**Level**	**Passivation** **Baths**	**Material**	**Concentration**	**Temperature** **°C**	**Time** **min**
		1	130.8	135.2	134	136.1	136.9
		2	131.9	136	137.1	135.1	134.3
		3	141				
		4	138.7				
		Delta	10.2	0.9	3.1	1	2.5
		Rank	1	5	2	4	3

**Table 7:** Gray relational analysis for I_corr_ in H_2_SO_4_ and NaCl solutions.

S. No	Normalized Values		Deviation Sequence		GRC		GRG	S/N GRG	Rank
	H_2_SO_4_	NaCl	H_2_SO_4_	NaCl	H_2_SO_4_	NaCl			
1	0.802	0.796	0.198	0.204	0.716	0.710	0.713	−2.93	8
2	0.933	0.278	0.067	0.722	0.882	0.409	0.646	−3.80	11
3	0.819	0.886	0.181	0.114	0.735	0.814	0.774	−2.22	7
4	0.462	0.823	0.538	0.177	0.482	0.738	0.610	−4.30	12
5	0.816	0.000	0.184	1.000	0.731	0.333	0.532	−5.48	15
6	0.988	0.882	0.012	0.118	0.977	0.809	0.893	−0.98	3
7	0.699	0.826	0.301	0.174	0.624	0.742	0.683	−3.31	10
8	0.947	0.941	0.053	0.059	0.904	0.895	0.900	−0.92	2
9	0.906	0.961	0.094	0.039	0.841	0.927	0.884	−1.07	4
10	0.478	0.960	0.522	0.040	0.489	0.925	0.707	−3.01	9
11	0.002	0.913	0.998	0.087	0.334	0.852	0.593	−4.54	13
12	0.705	0.991	0.295	0.009	0.629	0.982	0.806	−1.88	6
13	0.830	0.984	0.170	0.016	0.747	0.970	0.858	−1.33	5
14	1.000	1.000	0.000	0.000	1.000	1.000	1.000	0.00	1
15	0.000	0.793	1.000	0.207	0.333	0.707	0.520	−5.68	16
16	0.151	0.827	0.849	0.173	0.371	0.743	0.557	−5.08	14

**Table 8:** Average of parameters GRG.

Level	Passivation Baths	Material	Concentration	Temperature	Time
1	0.6858	0.7792	0.6960	0.7018	0.6949
2	0.7520	0.6804	0.7636	0.7578	0.7648
3	0.7475				
4	0.7339				
Delta	0.0662	0.0988	0.0677	0.0559	0.0699
Rank	4	1	3	5	2

**Table 9:** ANOVA for individual response.

	**Source**	**DOF**	**Adj SS**	**Adj MS**	**F-Value**	***p*** **-Value**	**% Contribution**
1% *v*/*v* H_2_SO_4_	Passivation baths	3	143.87	47.96	1.57	0.271	16.98
	Material	1	277.25	277.25	9.07	0.017	32.73
	Concentration	1	17.03	17.03	0.56	0.477	2.01
	Temperature	1	116.08	116.08	3.8	0.087	13.70
	Time	1	48.19	48.19	1.58	0.245	5.69
	Error	8	244.43	30.55	---	---	---
	Total	15	846.85	---	---	---	---
	**Source**	**DOF**	**Adj SS**	**Adj MS**	**F-Value**	***p*** **-Value**	**% Contribution**
3.5% *w*/*v* NaCl	Passivation baths	3	298.708	99.569	1.86	0.215	37.39
	Material	1	3.007	3.007	0.06	0.819	0.37
	Concentration	1	38.402	38.402	0.72	0.422	4.80
	Temperature	1	4.034	4.034	0.08	0.791	0.50
	Time	1	25.657	25.657	0.48	0.509	3.21
	Error	8	428.937	53.617	---	---	---
	Total	15	798.746	---	---	---	---

**Table 10:** ANOVA for GRG.

	DOF	Adj SS	Adj MS	F-Value	*p*-Value	% Contribution
Passivation baths (A)	3	0.011027	0.003676	0.21	0.881	3.31
Material (B)	1	0.039083	0.039083	2.27	0.373	11.75
Concentration (C)	1	0.018323	0.018323	1.06	0.490	5.51
Temperature (D)	1	0.012522	0.012522	0.73	0.551	3.76
Time (E)	1	0.019534	0.019534	1.13	0.480	5.87
Passivation baths × Material (A × B)	3	0.129055	0.043018	2.5	0.428	38.81
Passivation baths × Time (A × E)	3	0.085598	0.028533	1.66	0.506	25.74
Material × Time (B × E)	1	0.000122	0.000122	0.01	0.947	0.036
Error	1	0.017239	0.017239	---	---	---
Total	15	0.332502	---	---	---	---

**Table 11:** Results of confirmation test.

Response Variables	Initial Conditions	Optimal Condition	
		Predicted	Experimental
Level	A1B2C1D1E2	A2B1C1D2E2	A2B1C1D2E2
I_corr_ in H_2_SO_4_ (A/cm^2^)	5.364 × 10^−7^		5.646 × 10^−8^
I_corr_ in NaCl (A/cm^2^)	2.246 × 10^−7^		7.859 × 10^−8^
GRA	0.610	0.8982	0.950

**Table 12:** Results of confirmation test in H_2_SO_4_ solution.

S. No.	E_corr_ (V)	E_A–C_ (V)	E_pit_ (V)	I_corr_ (A/cm^2^)	Hysteresis
A1B2C1D1E2	−0.195	0.679	-	5.364 × 10^−7^	- Negative
A2B1C1D2E2	−0.155	0.715	0.865	5.646 × 10^−8^	+ Positive
CUSTOM 450	−0.284	0.571	-	3.623 × 10^−6^	- Negative
AM 350	−0.253	0.676	0.869	1.316 × 10^−6^	+ Positive

**Table 13:** Results of confirmation test in NaCl solution.

S. No.	E_corr_ (V)	E_A–C_ (V)	E_pit_ (V)	i_corr_ (A/cm^2^)	Hysteresis
A1B2C1D1E2	−0.244	−0.261	0.298	2.246 × 10^−7^	positive
A2B1C1D2E2	−0.234	−0.256	0.071	7.859 × 10^−8^	positive
CUSTOM 450	−0.308	−0.256	0.116	8.697 × 10^−7^	positive
AM 350	−0.376	−0.261	0.198	2.866 × 10^−7^	positive

## Data Availability

The original contributions presented in this study are included in the article. Further inquiries can be directed to the corresponding authors.

## Abbreviations

The following abbreviations are used in this manuscript:

PHSS	Precipitation-hardening stainless steels
GRA	Gray relational analysis
ANOVA	Analysis of variance
% *v*/*v*	Volume/volume concentration
°C	Celsius degrees
min	Minutes
H_2_SO_4_	Sulfuric acid
NaCl	Sodium chloride
L_n(m^k^)	Orthogonal arrays
*n*	Number of experiments
*m*	Number of levels of each factor
*k*	Maximum number of factors that can be studied
S/N	Signal-to-noise ratio
R_a_	Surface roughness
F value	Ratio between the variance explained by the factor and the variance of the error
*p*	Significant factors
GRCs	Gray relational coefficients
GRGs	Gray relational grades
GTAW	Gas tungsten arc welding
HVs	Hardness vickers
cm	centimeter
wt.%	Percentage by weight
C_6_H_8_O_7_	Citric acid
% *w*/*v*	Weight/volume concentration
DOE	Design of experiments
A	Bath chemical composition
B	Alloy chemical composition
C	Bath concentration
D	Temperature
E	Time
MOA	Mixed orthogonal array
DOFs	Degrees of freedom
I_corr_	Corrosion current density
OAs	Orthogonal arrays
CPPC	Cyclic potentiodynamic polarization curves
SCE	Saturated calomel electrode
OCP	Open circuit potential
*y_i_*	Observed data point
SS Factor	Sum of squares for each factor
SST	Total sum of squares for all factors
*x_i_*(*k*)	Normalized value
max*y_i_*(*k*)	The maximum values
min*y_i_*(*k*)	The minimum values
*k*-th	Response
∆_0_*i*(*k*)	Deviation sequence for the *i*-th trial at the *k*-th response variable
*x*_0_(*k*)	Reference (or ideal) value for the *k*-th response
*x_i_*(*k*)	Normalized value for the *i*-th trial and *k*-th response variable
*ξ_i_*(*k*)	GRC for the *i*-th response at the *k*-th condition
∆_min_	Minimum deviation sequences
∆_max_	Maximum deviation sequences
*r*	Distinguishing coefficient
*γ*i	GRG for the *i*-th experiment
S. No.	Sample number
*n_m_*	Represents the overall mean of the S/N or GRG
*n_j_*	Represents the mean S/N or GRG at the optimized level
*n*	Denotes the number of significant passivation process parameters
E_A–C_	Active–passive transition at positive potentials
E_corr_	Corrosion potential
E_pit_	Pitting potential
Cl^−^	Chloride ion
R_ct_	Charge transfer resistance
C_dl_	Double layer capacitance
EIS	Electrochemical impedance spectroscopy
